# Temporal Vestibular Deficits in *synaptojanin 1* (*synj1*) Mutants

**DOI:** 10.3389/fnmol.2020.604189

**Published:** 2021-01-18

**Authors:** Yan Gao, Teresa Nicolson

**Affiliations:** Department of Otolaryngology, Stanford University, Stanford, CA, United States

**Keywords:** synaptojanin, ribbon synapse, hair cell, zebrafish, vestibulospinal reflex, synaptic vesicle (SV)

## Abstract

The lipid phosphatase *synaptojanin 1 (synj1)* is required for the disassembly of clathrin coats on endocytic compartments. In neurons such activity is necessary for the recycling of endocytosed membrane into synaptic vesicles. Mutations in zebrafish *synj1* have been shown to disrupt the activity of ribbon synapses in sensory hair cells. After prolonged mechanical stimulation of hair cells, both phase locking of afferent nerve activity and the recovery of spontaneous release of synaptic vesicles are diminished in *synj1* mutants. Presumably as a behavioral consequence of these synaptic deficits, *synj1* mutants are unable to maintain an upright posture. To probe vestibular function with respect to postural control in *synj1* mutants, we developed a method for assessing the vestibulospinal reflex (VSR) in larvae. We elicited the VSR by rotating the head and recorded tail movements. As expected, the VSR is completely absent in *pcdh15a* and *lhfpl5a* mutants that lack inner ear function. Conversely, *lhfpl5b* mutants, which have a selective loss of function of the lateral line organ, have normal VSRs, suggesting that the hair cells of this organ do not contribute to this reflex. In contrast to mechanotransduction mutants, the *synj1* mutant produces normal tail movements during the initial cycles of rotation of the head. Both the amplitude and temporal aspects of the response are unchanged. However, after several rotations, the VSR in *synj1* mutants was strongly diminished or absent. Mutant *synj1* larvae are able to recover, but the time required for the reappearance of the VSR after prolonged stimulation is dramatically increased in *synj1* mutants. Collectively, the data demonstrate a behavioral correlate of the synaptic defects caused by the loss of *synj1* function. Our results suggest that defects in synaptic vesicle recycling give rise to fatigue of ribbons synapses and possibly other synapses of the VS circuit, leading to the loss of postural control.

## Introduction

Upon fusion of synaptic vesicles at synapses, retrieval and internalization of plasma membrane requires clathrin-mediated or bulk endocytosis (Milosevic, 2018; Chanaday et al., 2019; Bonnycastle et al., 2020). After bulk endocytosis occurs, the clathrin-associated machinery is also needed at a later step to recycle large endocytic compartments into small vesicles (Kononenko and Haucke, 2015). Once formed, clathrin coated vesicles are relieved of their surface coats via the actions of synaptojanin lipid phosphatases. These enzymes remove phosphate groups from phosphoinositide lipids such as PI(4,5)P2, which promotes the release of the clathrin protein complex (Mani et al., 2007). Uncoated vesicles can then rejoin the pool of synaptic vesicles for another cycle of neurotransmitter release.

Synaptojanin 1 contains two active sites, a SAC1 domain and a second 5′ phosphatase domain (McPherson et al., 1996; Guo et al., 1999). The 5′ phosphatase domain targets plasma membrane PIP2 and selectively mediates uncoating of synaptic vesicles (Cremona et al., 1999). Such activity is essential for survival in vertebrates. In humans, loss of both phosphatase domains causes severe defects such as untreatable epilepsy and neural decline in the first decade of life that leads to early mortality (Dyment et al., 2015; Hardies et al., 2016; Al Zaabi et al., 2018). In contrast, missense mutations in *SYNJ1* generally induce a slower pace of neural degeneration in humans. For example several mutations in *SYNJ1* have been identified in patients with early onset Parkinson's disease *PARK20* (Krebs et al., 2013; Quadri et al., 2013; Olgiati et al., 2014; Kirola et al., 2016). In *PARK20* patients, mutations such as R258Q occur in the SAC1 domain, which is thought to be mainly active at cytosolic membrane compartments such as the Golgi and endosomes where its lipid substrates are enriched (Di Paolo and De Camilli, 2006). Recent evidence has shown that the R258Q mutation is associated with enlarged early endosomes in human cells (Fasano et al., 2018). In addition, a mouse knock-in (KI) of R258Q was shown to display fewer synaptic vesicles and an increase in coated vesicles in nerve terminals (Cao et al., 2017). The mouse KI neurons also have a slower rate of endocytosis in neurons. Thus, the SAC1 domain of SYNJ1 plays an underappreciated role in plasma membrane recycling. All in all both early and late forms of Parkinson's disease primarily affect motor functions, leading to tremors, slowed movements and impaired balance (Postuma et al., 2015), but abnormal posture and non-motor abnormalities can also be present even before the onset of motor symptoms (Jellinger, 2017; Choi et al., 2020).

Animal models of *synj1* function have offered some insight into the role of membrane recycling in the nervous system. In worms and flies, endocytosis is defective and the number of synaptic vesicles is reduced (Harris et al., 2000; Verstreken et al., 2003; Dickman et al., 2005). Mouse knockouts of *Synj1* die perinatally due to a failure to suckle or thrive (Cremona et al., 1999). Nevertheless, experiments with mouse *Synj1*^−/−^ brain extracts established the importance of SYNJ1 in lipid phosphatase activity and uncoating of membranes (Cremona et al., 1999). *synj1* was also shown to be vital for the function of the peripheral nervous system in zebrafish. Both visual and vestibular deficits have been attributed to nonsense mutations in *synj1* (Van Epps et al., 2004; Trapani et al., 2009). These deficits affect a unique synaptic structure that is present in both photoreceptors and sensory hair cells, the ribbon synapse. In the cone photoreceptors of *synj1* mutants, ribbon synapses do not develop properly and ribbon bodies are free floating rather than attached to the active zone (Van Epps et al., 2001). As a consequence, mutant *synj1* larvae are blind (Allwardt et al., 2001). In contrast to cone photoreceptors, ribbon synapses in hair cells are present at presynaptic sites in mutant *synj1* larvae, but there are manifestations of membrane defects such as a reduction in the number of synaptic vesicles near the ribbons and blebbing of the plasma membrane at active zones. These defects suggest that endocytosis of plasma membrane is impaired. Interestingly, plasma membrane blebbing is rescued if presynaptic calcium influx and the resulting exocytosis of synaptic vesicles is blocked genetically in a *cav1.3a* and *synj1* double mutant (Trapani et al., 2009). In addition, hair-cell synapses in *synj1* mutants are unable to cope with higher frequency or prolonged stimuli, resulting in the release of neurotransmitter that is out of phase with mechanical stimuli and slower rates of replenishment of vesicles for spontaneous release. In terms of inner ear function, *synj1* mutants have moderately to severely reduced vestibular induced eye movements, indicating that transmittal of vestibular cues to the brain is dampened or absent (Trapani et al., 2009). Such a dearth of vestibular information could account for the balance and postural defects in *synj1* mutants, however, vestibular-evoked motor function has not been explored. Whether there is a behavioral correlate of synaptic fatigue due to the loss of *synj1* function remains to be determined.

Given the impairment of posture and balance in Parkinson's patients and zebrafish *synj1* mutants, we sought to characterize the role of *synj1* in postural control to gain a better understanding of the vestibular deficits. To assess potential defects in vestibular input and motor output in *synj1* mutants, we developed a method for quantifying the VSR in zebrafish larvae. The vestibulospinal circuitry is highly conserved in vertebrates and in its simplest form consists of a three neuron arc—afferent neurons that innervate vestibulospinal neurons, which in turn activate motor neurons that innervate head or trunk muscles (Straka and Baker, 2013; Bagnall and Schoppik, 2018). Movements produced by the neck and trunk muscles then adjust or correct the position of the body relative to gravity. In developing zebrafish larvae, the anterior otolith (and presumably the downstream VS neural circuit) mediates self-righting behavior (Bagnall and McLean, 2014). Righting reflexes are absent or diminished in zebrafish vestibular mutants such those carrying mutations in *synj1* (Trapani et al., 2009; Nicolson, 2015, 2017). In this study, we elicited trunk movements by rotating the head with a yaw stimulus. We validated our method by testing mutants that lacked inner ear function. Our results indicate that *synj1* mutants have a normal VSR that quickly declines after repetitive stimulation. Such a decline is likely due to synaptic fatigue at ribbon synapses.

## Materials and Methods

### Zebrafish Husbandry and Use

Zebrafish (*Danio rerio*) were maintained and bred using standard procedures (Westerfield, 1993). Animal research complied with guidelines of Laboratory Animal Care and Use stipulated by Stanford University. The zebrafish mutant alleles used for this study were *pcdh15a*^*th*263*b*^, *lhfpl5a*^*tm*290*d*^, *lhfpl5b*^*vo*35^, *synj1*^*Q*296*X*^ (Seiler et al., 2005; Trapani et al., 2009; Erickson et al., 2019). All homozygous and wild type sibling larvae were tested at 5 or 6 days post-fertilization (dpf) before sex determination.

### Genotyping

Standard genomic PCR followed by Sanger sequencing was used to identify the *pcdh15a*^*th*263*b*^, *lhfpl5a*^*tm*290*d*^, and *lhfpl5b*^*vo*35^ alleles. The following primers were used for genotyping:

*pcdh15a*^*th*263*b*^: Fwd – AGGGACTAAGCCGAAGGAAG, Rvs – CACTCATCTTCACAGCCATACAG.
*lhfpl5a*^*tm*290*d*^: Fwd – GGACCATCATCTCCAGCAAAC, Rvs – CACGAAACATATTTTCACTCACCAG;
*lhfpl5b*^*vo*35^: Fwd – GCGTCATGTGGGCAGTTTTC, Rvs – TAGACACTAGCGGCGTTGC.

We also used FM 1–43 dye to identify *lhfpl5b*^*vo*35^ larvae. Because of the disruption of the mechanotransduction complex in lateral line hair cells, *lhfpl5b*^*vo*35^ larvae do not label with bath applied FM 1–43 (Erickson et al., 2019). *synj1*^*Q*296*X*^ alleles were identified by using kompetitive allele specific PCR (KASP) genotyping (LGC Genomics), which uses endpoint fluorescence detection to discriminate tagged alleles.

### VSR Test

The construction of VSR test device was described in Sun et al. (2018). We designed a new mounting chamber with a narrow slot to immobilize the head of larvae, which we inserted into a 5 mm wide slot on the chamber holder. The motor rocked the platform from −75° to + 75° at 0.53 Hz. The videos were generated at 30 frames per second (fps). Up to 1,280 frames of video recording (~42 s) were captured as an AVI file.

For mounting the larvae, we liquified 2% low melting agarose in E3 buffer at 42°C. Larvae were transferred into the mounting chamber and excess E3 buffer was removed. A drop of agarose liquid was added on top of each specimen. Then the larva was quickly adjusted to a dorsal-up position with the head was toward the narrow slot using a fine dissecting probe before the agarose solidified. After solidification, the agarose was gently removed around the trunk and pectoral fins. Approximately 20 μl of E3 media was added to the exposed trunk area. Larvae were kept in complete darkness during the experiment.

### Data Analysis and Quantification

We used a modified version of ZebraZoom software (courtesy of Mirat, 2020) written in Python 3.8 programming language to track tail movements during yaw stimulation. ZebraZoom tracks the movements of the trunk marked at regular intervals and calculates the real tail angle over time between the X axis and the axis formed by the tip of the tail and the center of the swimming bladder.

The following outputs were directly acquired from ZebraZoom (Supplementary ZebraZoom Script 1): tail angle; rolling median of tail angle (median of 5 neighboring tail angles); difference of tail angle and rolling median of tail angle (tail angle is calculated by subtracting the rolling median of tail angle); maximum tail angle of difference; normalized integral of difference; percentage of time above threshold (the percentage of frames for which the difference is above threshold, the default threshold is 5°). The tail angle of normal VSR ranges from 0 to 45°. Cycles that included random startle responses (tail movements larger than 45°) were manually excluded from analysis. The tail angle of startle response could hit more than 100°, which is much larger than the normal VSR. The random startle response rarely happened in the wild type siblings and mutants. All the output data were processed using Prism 8.4 (GraphPad software). We calculated the mean ± SEM for each genotype/dataset and used the two-way ANOVA with Benjamini-Hochberg correction. The Anderson-Darling, D'Agostino and Pearson, Shapiro-Wilk and Kolmogorov-Smirnov normality tests all yield values above 0.1, showing normal distribution of the data.

## Results

### The VSR in Zebrafish Larvae

The VSR has been studied in other species such as mice and cats (Andre et al., 2005; Di Bonito et al., 2015), but has not been described in zebrafish larvae. To characterize vestibular-evoked motor function in 5 day-old larvae, we established a method for quantification of the VSR. At this stage of development, the larvae are free swimming and reflexive movements of the trunk muscles in zebrafish larvae are predicted to arise in response to vestibular cues. Rotation of the head does not activate the immature semicircular canals at this stage of development, but rather leads to excitation of vestibular hair cells in the anterior maculae (Beck et al., 2004; Mo et al., 2010; Sun et al., 2018).

**Figure 1:**
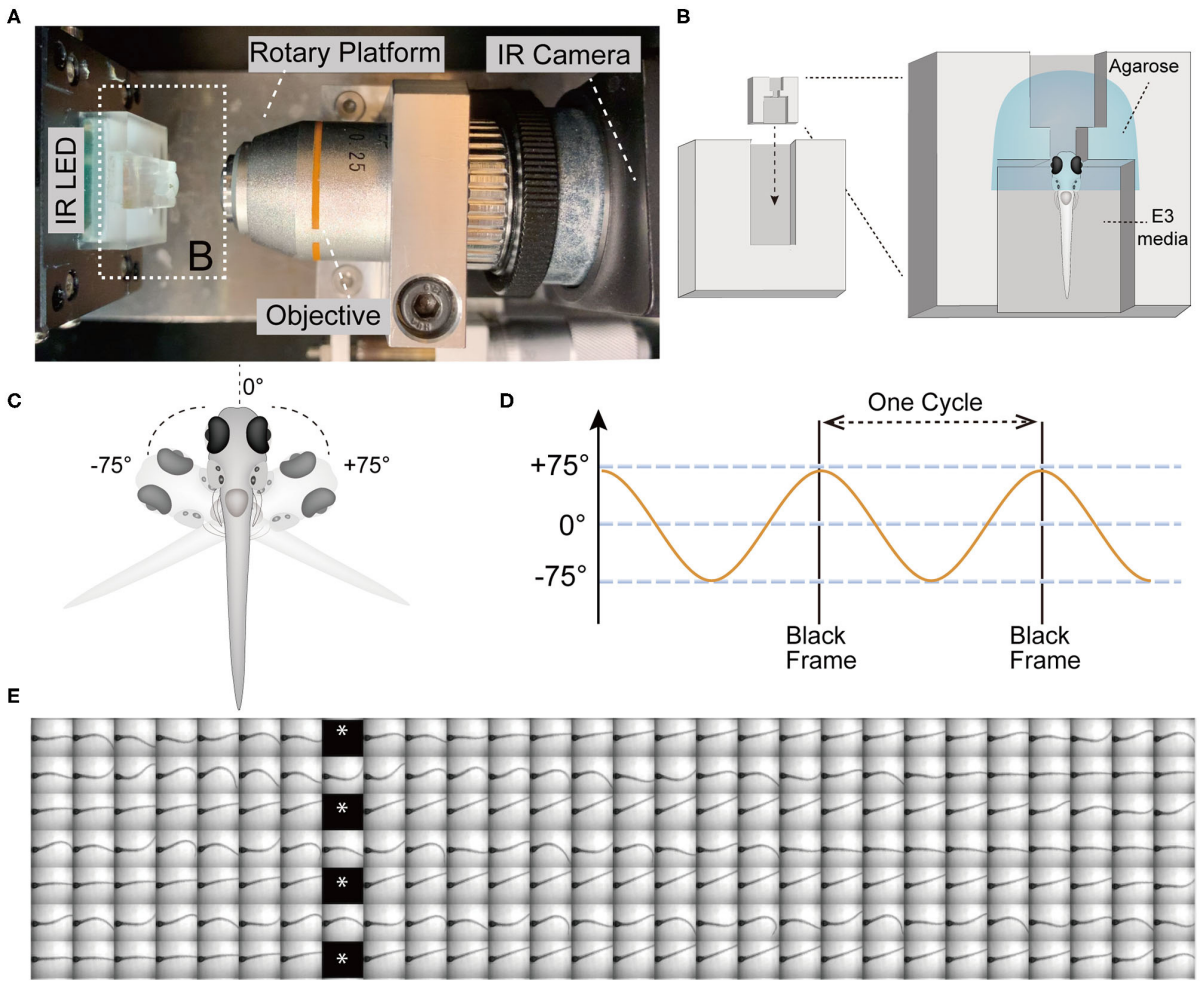
The VSR in zebrafish larvae at 5 days post-fertilization (5 dpf). **(A)** Infrared (IR) imaging acquisition system, which contains a rotary platform driven by a motor, an objective (10x), an IR LED beneath the chamber holder, and an IR camera. The white dotted box indicates the mounting chamber and chamber holder **(B)**. **(B)** Diagram of a vertically mounted larva in the chamber unit. The larval head is embedded in 2% low melting point agarose and the trunk and pectoral fins are free to move in the E3 media. **(C,D)** The test device provides a yaw stimulus at 0.53 Hz and rotation of the head ±75°. **(E)** Still frames of four contiguous cycles of the evoked tail responses in a wild type larva are shown. Asterisks indicate black frames.

For our experiments, we elicited tail movements in head-fixed larvae with a yaw stimulus in dark conditions and recorded responses using a device that was previously used to assess vestibular-induced eye movements in larvae (Sun et al., 2018). Briefly, the main components of the test device for infrared (IR) imaging include a rotary platform driven by a motor, an objective lens (10x), an IR light emitting diode (LED) to illuminate the larvae, an IR camera (Figure 1A), and a small chamber unit for mounting the larvae that can be inserted into the chamber holder (Figure 1B). In order to securely immobilize the head of larvae and the agarose around head, a narrow slot was added to the middle of the mounting chamber (Figure 1B). The larvae were mounted in a vertical position with the head up, and the tail and pectoral fins were freed from excess agarose (Figures 1B,C). Robust movements of the pectoral fins indicated that the larvae were active and presumably not injured during mounting. Previous studies have shown that zebrafish larvae alternate their pectoral fins in coordination with the body axis during slow swimming. The movements of the pectoral fins do not contribute to the production of thrust or stability but instead exchange fluid near the body for cutaneous respiration (Hale, 2014). In addition, pectoral fins are able to beat without the participation of the body axis (Green et al., 2011).

The test device provided a sinusoidal wave stimulus at 0.53 Hz. During each cycle, the IR LED paused (16 ms) to record one black frame when the rotary platform arrived at its limit at ±75° (Figures 1C–E). The black frames mark the beginning and end of one cycle, which lasts for 1.84 s and contains 56 frames (Figures 1D,E). Each black frame allowed us to track the cycles and quantify the recorded movements within a cycle (Figure 1E). We used a total of 21 cycles of yaw stimulation for our analyses.

### Tracking of Tail Movements Using ZebraZoom Program

**Figure 2:**
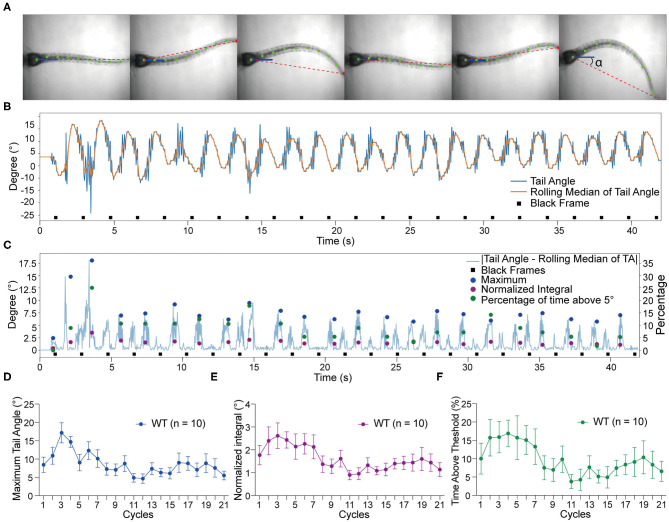
Tracking of tail movements using ZebraZoom software. **(A)** Six contiguous frames of tail movements from a wild type larva. ZebraZoom calculates the angle of the red dotted line and the horizontal line as real tail angle (α). **(B)** Representative tracking trace of tail movements from a wild type larva. **(C)** The output data of the maximum tail angle, normalized integral, and percentage of time above threshold are displayed as colored dots. **(D–F)** Group data of wild type larvae (*n* = 10) for maximum tail angle, normalized integral, and percentage of time above threshold. Means ± SEM are shown.

To quantify the vestibular-induced tail movements, we used a modified version of ZebraZoom program (Mirat et al., 2013) to analyze the videos recorded by the platform camera. For our analysis, ZebraZoom selects the initial axis of the trunk or “core position” as a reference point (blue line) and tracks 10 points along the tail (green dots) including the end point of the tail (red dot) to estimate the curvature of the tail and track activity (Figure 2A). Six contiguous frames of tail movements are shown from a representative wild type larva in Figure 2A and Supplementary Movie 1. The ZebraZoom program was used to calculate tail angle, which is the angle between the body axis and the line connecting the tip of the tail to the center of the swim bladder (Figure 2A). To assess movement artifacts, the rolling median of the tail angle was also determined as the median of the five neighboring tail angles. The rolling median represents the non-evoked swaying of the tail produced by the gravitational pull on the trunk of the larvae and the movement of liquid in the mounting chamber. A representative tracking trace of a wild type larva is shown in Figure 2B. In order to quantify the tail movements, the rolling median of tail angle was subtracted from the tail angle to obtain absolute values. We then determined the following metrics for each cycle of stimulation: (i) the maximum tail angle produced, (ii) the normalized integral of the movements, and (iii) the percentage of time spent above threshold, which was set at 5° for all experiments. The maximum tail angle reveals the amplitude of tail movements during testing, whereas the normalized integral and percentage of time above threshold reflect the level of activity in each cycle in terms of amplitude and duration. An example of each metric during the course of the experiment with the same larva as in Figure 2B is shown in Figure 2C. Due to technical reasons, we did not analyze the first and last cycles of each experiment, thus, only 21 cycles were quantified for each larva. The group data obtained with wild type larvae for maximum tail angle, normalized integral and percentage of time above threshold are shown in Figures 2D–F. In general, we observed a decrease in activity over the course of each trial, which may be due to adaptation to the stimulus.

### The VSR Is Absent in *pcdh15a* Mutants That Lack Inner Ear Function

**Figure 3:**
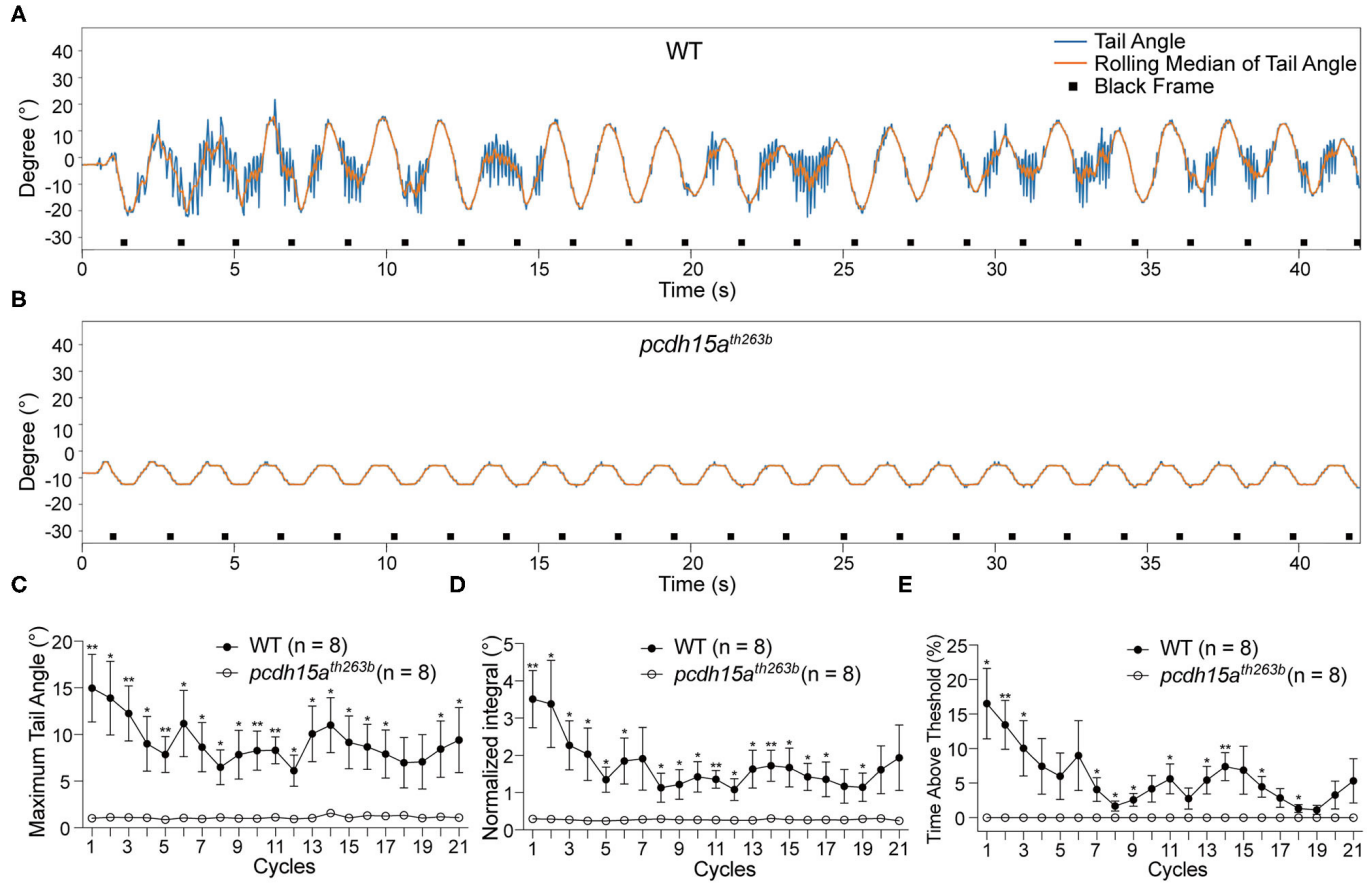
The VSR is absent in *pcdh15a* mutants that lack inner ear function. **(A,B)** Representative tracking traces of a wild type sibling and a *pcdh15a*^*th*263*b*^ mutant. **(C–E)** The maximum tail angle, normalized integral and percentage of time above threshold show significant differences between wild type siblings (*n* = 8) and *pcdh15a*^*th*263*b*^ mutants (*n* = 8). Mean ± SEM for each genotype was plotted and a two-way ANOVA with Benjamini-Hochberg correction was performed. *p < 0.05 and **p < 0.01.

To confirm that the tail movements are reflexes to vestibular cues rather than random movements, we performed the VSR test on mutants that lack inner ear and lateral-line organ function (Table 1). We first characterized the responses of *pcdh15a*^*th*263*b*^ larvae (Seiler et al., 2005; Maeda et al., 2017). Protocadherin 15 (Pcdh15) forms part of the tip link in hair cells, which is thought to gate mechanotransduction channels in sensory hair cells (Sakaguchi et al., 2009; Nicolson, 2017). In zebrafish, the *th263b* mutation is a null allele that leads to a severe truncation of Pcdh15a and abolishes mechanotransduction (Seiler et al., 2005; Maeda et al., 2017). During the yaw stimulation, *pcdh15a*^*th*263*b*^ larvae were completely unresponsive compared to wild type siblings (Figures 3A,B). No tail movements were observed beyond the rolling median of tail movements (representative trace shown in Figure 3B). The oscillation of the rolling median mimicked the sinusoidal pattern of the stimulus, indicative of a movement artifact induced by the platform. Consistent with the individual shown in Figure 3B, the metrics of the VSR of *pcdh15a*^*th*263*b*^ larvae were profoundly different compared to wild type siblings (Figures 3C–E). These results demonstrate that the function of sensory hair cells is vital for evoked tail movements.

**Table 1:** Alleles used in this study.

**Gene**	**Mutant alleles**	**Gene expression**	**Function**	**References**
*pcdh15a*	*th263b*	Inner ear, lateral line organ, brain	Hair-cell mechanotransduction	Sakaguchi et al., 2009; Maeda et al., 2017; Nicolson, 2017
*lhfpl5a*	*tm290d*	Inner ear	Hair-cell mechanotransduction	Nicolson et al., 1998; Erickson et al., 2019
*lhfpl5b*	*vo35*	Lateral line organ	Hair-cell mechanotransduction	Nicolson et al., 1998; Erickson et al., 2019
*synj1*	*Q296X*	Inner ear, lateral line organ, brain, retina	Synaptic vesicle recycling	Trapani et al., 2009

### The Lateral Line Organ Does Not Contribute to the VSR

Loss of *pcdh15a* causes dysfunction of both the inner ear and lateral line organ. Given the movement of the mounting media during the rotary stimulation, it was not clear whether the lateral line organ, which is comprised of clusters of sensory hair cells (neuromasts) at the surface of the head and trunk of zebrafish larvae, was also responding and signaling to the VS circuit.

To determine whether the lateral line organ provides sensory input, we tested *lhfpl5a*^*tm*290*d*^ and *lhfpl5b*^*vo*35^ mutants. At the larval stage, *lhfpl5a* and *lhfpl5b* are expressed in distinct populations of sensory hair cells (Maeda et al., 2017). *lhfpl5a* is expressed in the inner ear, but its expression is not detected in the neuromasts. In contrast, *lhfpl5b* is exclusively expressed in neuromasts, both on the trunk and head, and *lhfpl5b* transcripts are not detectable in the inner ear. Functional experiments have shown that *lhfpl5a* is required for auditory and vestibular hair-cell function, whereas *lhfpl5b* is required for hair-cell function in the lateral line organ (Erickson et al., 2019). However, the lateral line hair cells are still mechanically sensitive in *lhfpl5a* mutants (Nicolson et al., 1998; Erickson et al., 2019).

**Figure 4:**
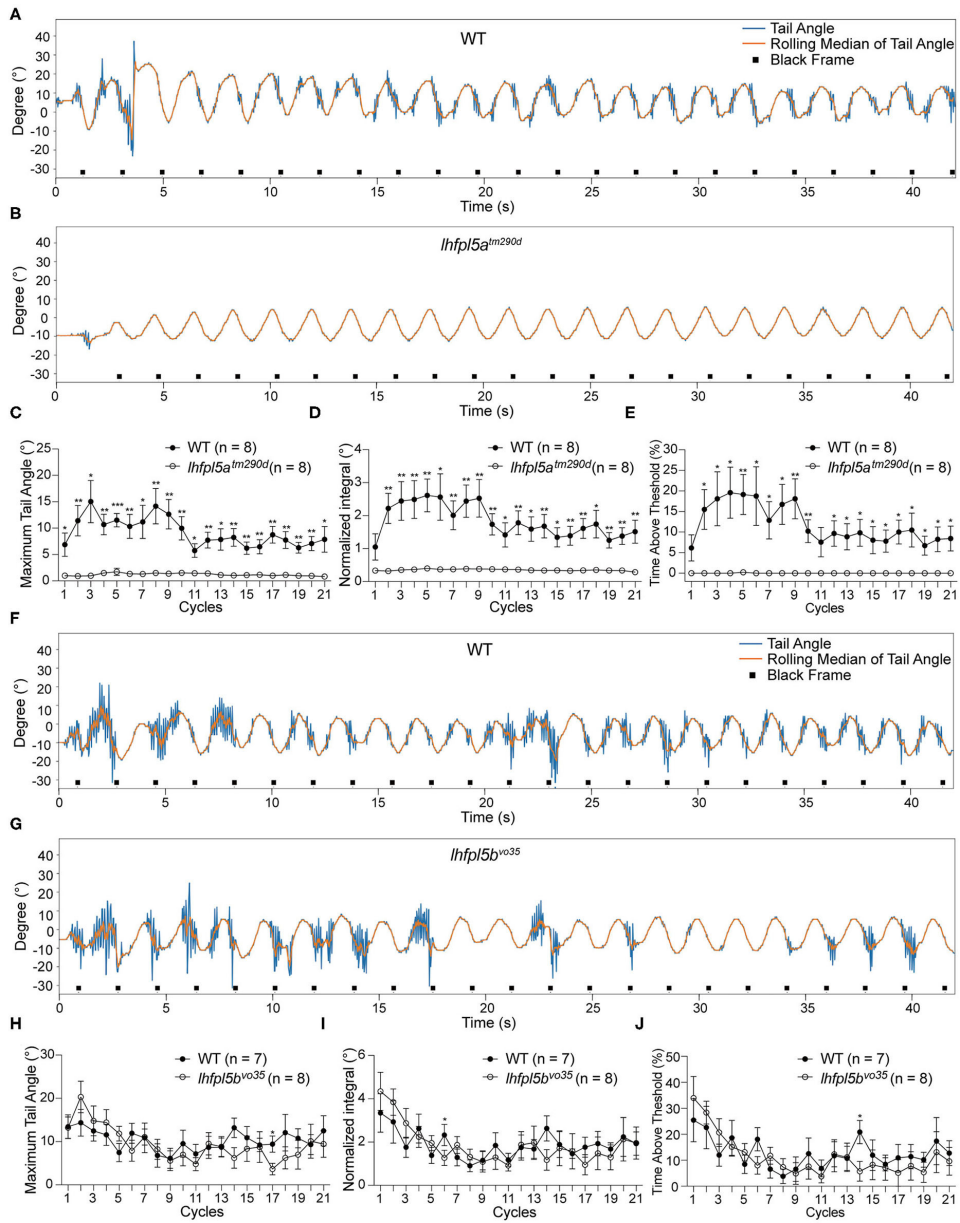
The larval lateral line organ does not contribute to the VSR. **(A,B)** Representative tracking traces of a wild type sibling and a *lhfpl5a*^*tm*290*d*^ mutant. **(C–E)** The maximum tail angle, normalized integral and percentage of time above threshold are significantly reduced in *lhfpl5a*^*tm*290*d*^ mutants (*n* = 8) compared to wild type siblings (*n* = 8). **(F,G)** Representative VSR tracking traces show comparable tail movements in a wild type sibling and a *lhfpl5b*^*vo*35^ mutant. **(H–J)** The maximum tail angle, normalized integral, and percentage of time above threshold are comparable between wild type siblings (*n* = 7) and *lhfpl5b*^*vo*35^ mutants (*n* = 8). Mean ± SEM for each genotype was plotted and a two-way ANOVA with Benjamini-Hochberg correction was performed. *p < 0.05; **p < 0.01; and ***p < 0.001.

We tested the VSR in *lhfpl5a*^*tm*290*d*^ mutants and wild type siblings. Representative tracking traces are shown in Figures 4A,B. Like the *pcdh15a* mutants, the VSR is absent in *lhfpl5a*^*tm*290*d*^ mutants. All metrics for the maximum of the tail angle (Figure 4C), normalized integral (Figure 4D), and percentage of time above threshold (Figure 4E) for *lhfpl5a*^*tm*290*d*^ mutants were profoundly reduced compared to wild type siblings. We next tested the VSR of *lhfpl5b*^*vo*35^ mutants (Figures 4F,G). In contrast to the *lhfpl5a* inner ear mutant, the tail movements of *lhfpl5b*^*vo*35^ mutants were robust and comparable to those observed in wild type siblings (Figures 4H–J). We observed only two cycles that were mildly yet statistically significantly (*p* < 0.05) reduced in the *lhfpl5b* mutants. Taken together, our results suggest that the VSR is dependent on the function of the larval inner ear, whereas the contribution by the lateral line organ is negligible for this reflex.

### Severe Decline of the VSR Over Time in *synj1* Mutants

**Figure 5:**
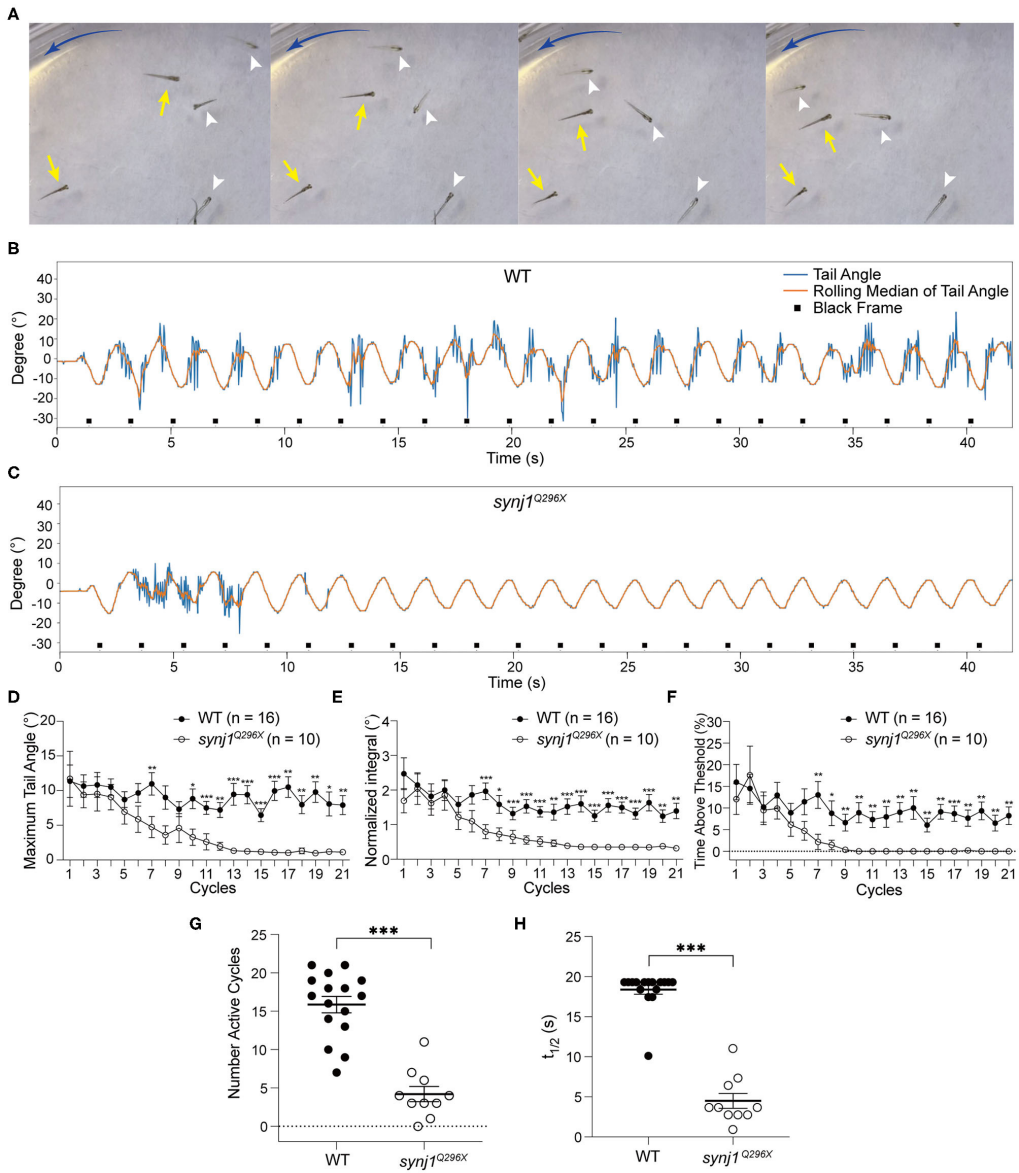
The VSR rapidly decreases over time in *synj1* mutants. **(A)** Impaired balance in *synj1*^*Q*296*X*^ mutants. The blue arc-shape arrow indicates the direction of the current. Yellow arrows indicate wild type siblings and white arrowheads indicate *synj1*^*Q*296*X*^ mutants. **(B,C)** Representative tracking trace of a wild type sibling and a *synj1*^*Q*296*X*^ larva. Note the tail movements during the initial cycles and then cessation of the reflex. **(D–F)** The maximum tail angle, normalized integral, and percentage of time above threshold show no significant difference during the initial cycles, but then dramatically decrease in *synj1*^*Q*296*X*^ larvae. **(G)** The number of active cycles (default threshold was 5°) were reduced in *synj1* mutants compared to wild type siblings. **(H)** The half time of the VSR in *synj1*^*Q*296*X*^ larvae was significantly less compared to wild type siblings. Mean ± SEM for each genotype was plotted and a two-way ANOVA with Benjamini-Hochberg correction was performed. *p < 0.05; **p < 0.01; and ***p < 0.001.

Similar to the *pcdh15a* and *lhfpl5a* mutants, *synj1*^*Q*296*X*^ larvae exhibit impaired balance, albeit to a lesser extent (Trapani et al., 2009). When challenged by swirling in a dish of E3 media, the postural defects of *synj1*^*Q*296*X*^ larvae are very pronounced, including floating along with the current in an upside-down position (Figure 5A). In contrast to *pcdh15a* and *lhfpl5a* mutants, mechanotransduction in sensory hair cells is normal in *synj1* mutants (Trapani et al., 2009). Instead, mutations in *synj1* lead to deficits in synaptic vesicle recycling and neurotransmitter release in hair cells. Presumably these deficits cause a reduction in vestibular-induced eye movements, suggests that signaling from the inner ear to brain is attenuated in *synj1* mutants (Trapani et al., 2009). To test this hypothesis and explore the basis of the postural defects, we assessed the VSR in *synj1* mutants.

In all experiments, the tracking traces showed that *synj1*^*Q*296*X*^ larvae produced robust tail movements during the first phase of stimulation compared to wild type siblings (Figures 5B,C). Moreover, the amplitude and activity level of the VSR in *synj1*^*Q*296*X*^ larvae were indistinguishable in the initial cycles (Figures 5D–F). However, we observed that the VSR in *synj1* mutants was strongly attenuated or completely disappeared over the course of the experiment (Figures 5D–F). The raw output data of the VSR in wild type siblings and *synj1*^*Q*296*X*^ larvae is shown in Supplementary Table 1. Overall the number of active cycles (default threshold was 5°) were starkly reduced in *synj1*^*Q*296*X*^ larvae compared to wild type siblings (Figure 5G). To determine whether there was a gene dosage effect, we separated the data for wild type siblings into homozygous wild type and heterozygous *synj1*^*Q*296*X*^ larvae, however, we did not observe a significant difference in responses between the two genotypes (Supplementary Figure 1). To assess the rate of decline in activity, we determined the time at which the response was reduced to half using the normalized integral values. The half time of the VSR in *synj1* mutants was much shorter compared to wild type siblings (Figure 5H). These data suggest that the synaptic defects caused by the loss of *synj1* function affect motor output in a temporal manner, showing a rapid decline over time.

### Recovery Time Is Delayed in *synj1* Mutants

Our previous study of *synj1* mutants revealed that prolonged mechanical stimulation of hair cells resulted in a long delay in the return of neurotransmitter release (Trapani et al., 2009). The decline of the VSR over time in *synj1* mutants is consistent with a vesicle recycling defect and synaptic fatigue. To further examine the temporal aspects of the rapid decline of the VSR, we characterized the duration of pauses between activity.

**Figure 6:**
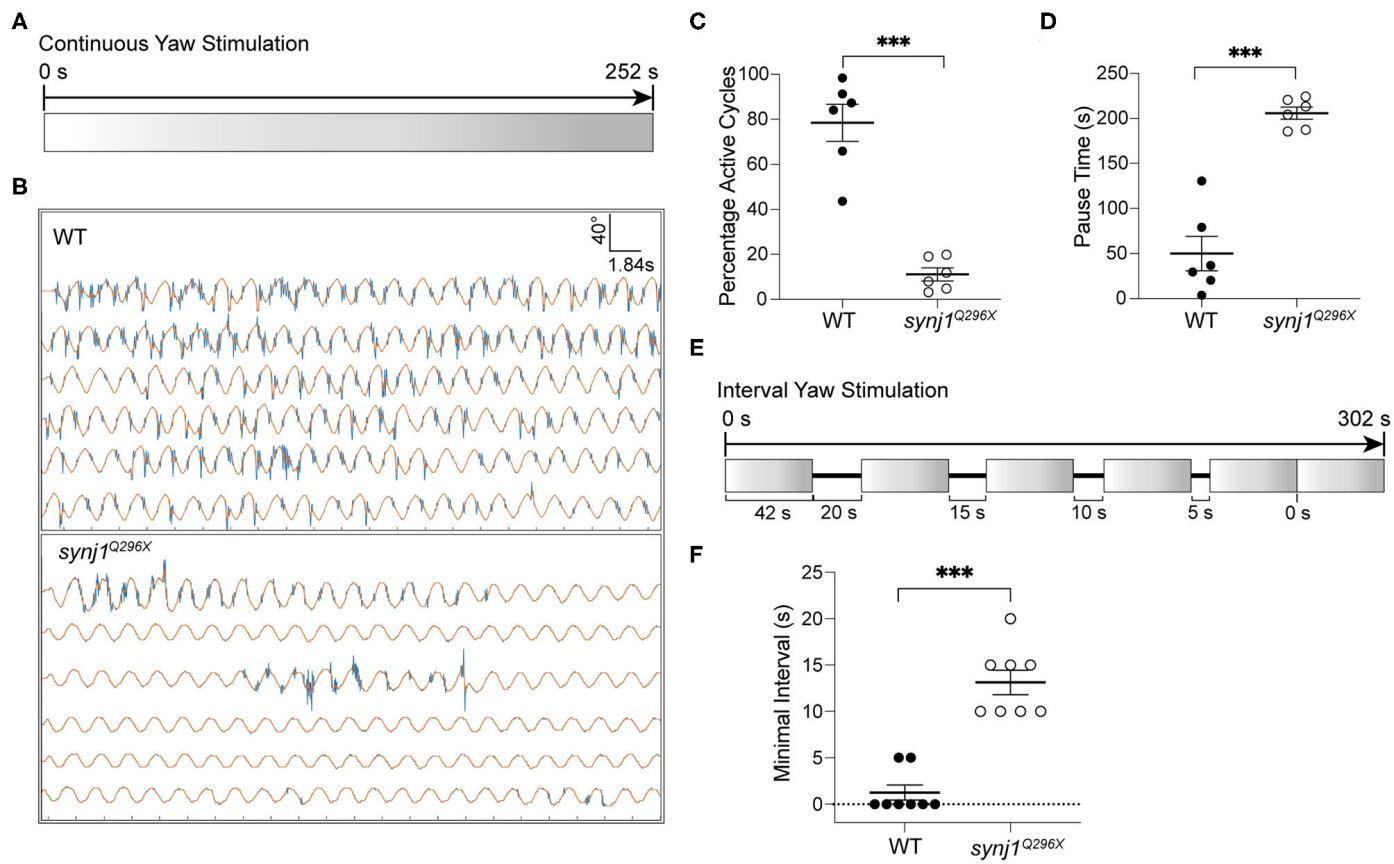
Longer pauses in activity and increased recovery time required for the resumption of the VSR in *synj1* mutants. **(A)** Diagram of prolonged yaw stimulation (252 s). **(B)** Representative tracking traces of responses. Note the paucity in activity in the mutant. **(C,D)** Percentage of active cycles and duration of pauses. *synj1*^*Q*296*X*^ larvae display fewer active cycles and much longer recovery periods during prolonged stimulation. **(E)** Diagram of yaw stimulation with decreasing rest intervals. Six stimuli were given with rest periods in between each stimulus lasting 0–20 s (42 s per stimulus, 302 s in total). **(F)** The minimal time interval required to resume VSR activity. Mean ± SEM for each genotype was plotted and a two-way ANOVA with Benjamini-Hochberg correction was performed. ***p < 0.001.

We performed either extended trials or yaw stimulations with defined intervals to examine the length of pauses or recovery time required before resumption of tail movements. For extended stimulation, the larvae were subjected to prolonged rotary stimulation lasting over 4 min (Figure 6A). The tracking traces of tail movements revealed short pauses of activity in wild type larvae, whereas strikingly long pauses were observed for mutant larvae (Figure 6B). Overall, *synj1* mutants were active in far fewer cycles and had much longer pauses during extended stimulation (Figures 6C,D). For the trials with defined intervals, the larvae were given six rotary stimuli (42 s each) with varying rest periods (0–20 s) in between each stimulus as shown in Figure 6E. The minimal time interval required for recovery of VSR activity for *synj1* mutants ranged from 10 to 20 s, whereas wild type siblings maintained VSR activity throughout the trial and required no additional recovery time (Figure 6F). These results indicate that *synj1* mutants require substantially more recovery time compared to wild type siblings. The delay in recovery supports the idea that *synj1* mutants have fewer synaptic vesicles available for neurotransmitter release due to a defect in vesicle recycling.

## Discussion

Deficits in the transmission or processing of information are a common occurrence in neural disease. The role of synaptic plasticity and fatigue in pathology is thought to be a prominent one in neuropsychological disease and the loss of synaptic transmission may be a contributing factor to neurodegeneration (Crabtree and Gogos, 2014; Bonnycastle et al., 2020). The ability to assess the flow of information through a neural circuit can be crucial for understanding neural dysfunction.

In terms of sensory processing, evaluating the integration of sensory input and motor output can be key to identifying defects in information flow. Here we describe a method for quantifying a highly conserved behavioral reflex, the VSR, in zebrafish larvae. By rotating the head and imaging and quantifying tail movements in 5 dpf larvae, we were able to gauge the differences in tail bends and the level of activity in several zebrafish mutants that have severe or moderate deficits in hair-cell function. We observed that the complete loss of hair-cell function in larvae abrogated the VSR, validating our experimental set up. Taking advantage of the gene duplication of *lhfpl5* and the selective expression pattern of each duplicate (Erickson et al., 2019), we determined that the VSR is dependent on the function of the larval inner ear and that the lateral line organ does not appreciably contribute to this response.

In contrast to mutants that lack inner ear function and do not respond to rotation of the head, *synj1*^*Q*296*X*^ larvae had initially normal responses to vestibular cues. This initial response is consistent with our previous report on *synj1* mutants showing that synaptic transmission is not significantly affected at the outset when lateral-line hair cells are mechanically stimulated at 60 Hz (Trapani et al., 2009). The flow of information from mutant *synj1* lateral-line hair cells to afferent neurons is comparable to the wild type transfer of input, suggesting that mutant synapses can initially keep pace with the demands. However, upon prolonged stimulation of hair cells, afferent neurons that innervate lateral-line hair cells show a delay in spiking in *synj1* mutants, resulting in a decrease in phase locking with the mechanical stimulation of the hair bundle (Trapani et al., 2009). With respect to our VSR experiments, the frequency of stimulation is much lower (0.53 Hz). Vestibular hair cells are specialized for detecting low frequencies that are more relevant to head rotations and changes in linear acceleration. In contrast, lateral line hair cells can detect fast water flow and are sensitive to a very broad range of frequencies (1–150 Hz) (Bleckmann, 2008). Whether a defect in phase locking occurs in vestibular hair cells in *synj1* mutants remains to be determined, nonetheless, synaptic fatigue appears to be common to both types of hair cells. Upon prolonged mechanical stimulation, recovery of the spontaneous release of synaptic vesicles is greatly slowed in lateral-line hair cells. Moreover, the number of synaptic vesicles at hair-cell ribbon synapses is reduced in *synj1* mutants, whereas coated vesicles are increased (Trapani et al., 2009). Similar delays in recovery from synaptic depression and in synaptic vesicle reformation were reported in *Synj1*^−/−^ brain slices or cultured neurons (Cremona et al., 1999; Kim et al., 2002). In the present study we observed that after several cycles of rotation of the head, *synj1* mutants stopped responding with tail movements, suggesting that neurotransmitter release ceases due to the lack of readily releasable vesicles. Furthermore, the recovery time of the VSR after prolonged rotatory stimulation was on the order of several minutes, similar to the time scale required for spontaneous activity to return to lateral-line hair cells after prolonged mechanical stimulation. The steep rundown in neurotransmitter release may account for the balance defects in *synj1* mutant larvae, particularly when they are challenged with prolonged vestibular stimulation. Whether the rundown in neurotransmitter release occurs mainly in hair cells, which experience high demands in terms of exocytosis and membrane turnover, and not in other neurons comprising the VS circuitry will require further studies in conditional knock outs. The vestibular deficits in *synj1* mutants may indeed be due to the combined vesicle recycling defects at the first order and higher order synapses of the vestibular system.

Studies of the genetic basis of Parkinson's disease have established a strong link between abnormal intracellular vesicle trafficking and pathogenesis in the brain (Ebanks et al., 2019). It is notable that both *PARK20* human patients and R258Q KI mice have impaired balance (Cao et al., 2017), which is a shared feature with zebrafish *synj1* mutants. Hair cells of the inner ear express *synj1* at low levels in zebrafish (Trapani et al., 2009), but it is not clear if this is the case in other species. In terms of transcripts, some RNAseq datasets show that *Synj1* is expressed in the hair cells of other species, whereas others do not (gEAR database, unpublished observations, gEAR, 2020). The conflicting results may be due to the low expression of this gene in hair cells. Nonetheless, it is worth considering the possibility that the balance deficits in mice and humans with *PARK20* mutations are due in part to a peripheral defect, i.e., defective synaptic transmission at hair-cell synapses. Alternatively, synaptic fatigue in higher order synapses in the vestibulospinal circuit may be a general feature of *PARK20* that could account for impaired balance. Indeed, there is mounting evidence that vestibular-evoked myogenic potentials are abnormal in a subset of Parkinson's patients, and intriguingly, that vestibular stimulation offers some relief for patients, suggesting that there is link between postural instability and vestibular dysfunction (Smith, 2018), although more studies are needed.

In summary, we have developed and validated a method for testing the VSR in zebrafish larvae. Defining the various features of motor output evoked by vestibular cues in terms of magnitude and temporal aspects will aid in understanding defects in postural control. With regard to the balance defects in *synj1* mutants, our results demonstrate the existence of a behavioral correlate of synaptic fatigue. Such functional synaptic defects are the potential basis for other types of neural dysfunction and our study provides insights into the loss of vestibular function when synaptic vesicle recycling is attenuated.

## Data Availability Statement

The raw data supporting the conclusions of this article will be made available by the authors, without undue reservation.

## Ethics Statement

This animal study was reviewed and approved by Stanford University APLAC.

## Author Contributions

YG and TN conceived and designed the study and wrote the manuscript. YG collected and analyzed the data. All authors contributed to the article and approved the submitted version.

## Conflict of Interest

The authors declare that the research was conducted in the absence of any commercial or financial relationships that could be construed as a potential conflict of interest.

## Supplementary Material

Supplementary Figure 1 Lack of a gene dosage effect in *synj1*^*Q*296*X*^ sibling cohorts. Comparison of heterozygous *synj1*^*Q*296*X*^ wild type siblings vs. homozygous wild type siblings does not reveal significant differences in performance. **(A–D)** The maximum tail angle, normalized integral, percentage of time above threshold, and number of active cycles. Mean ± SEM for each genotype was plotted and a two-way ANOVA with Benjamini-Hochberg correction was performed for the wild type larvae. Mutant data points were added for comparison.
