# Supplementary figures and images for: Temporal Vestibular Deficits in synaptojanin 1 (synj1) Mutants
Source: Front Mol Neurosci. 2021 Jan 18;13:604189. doi: 10.3389/fnmol.2020.604189 (PMC7874208; doi:10.3389/fnmol.2020.604189)

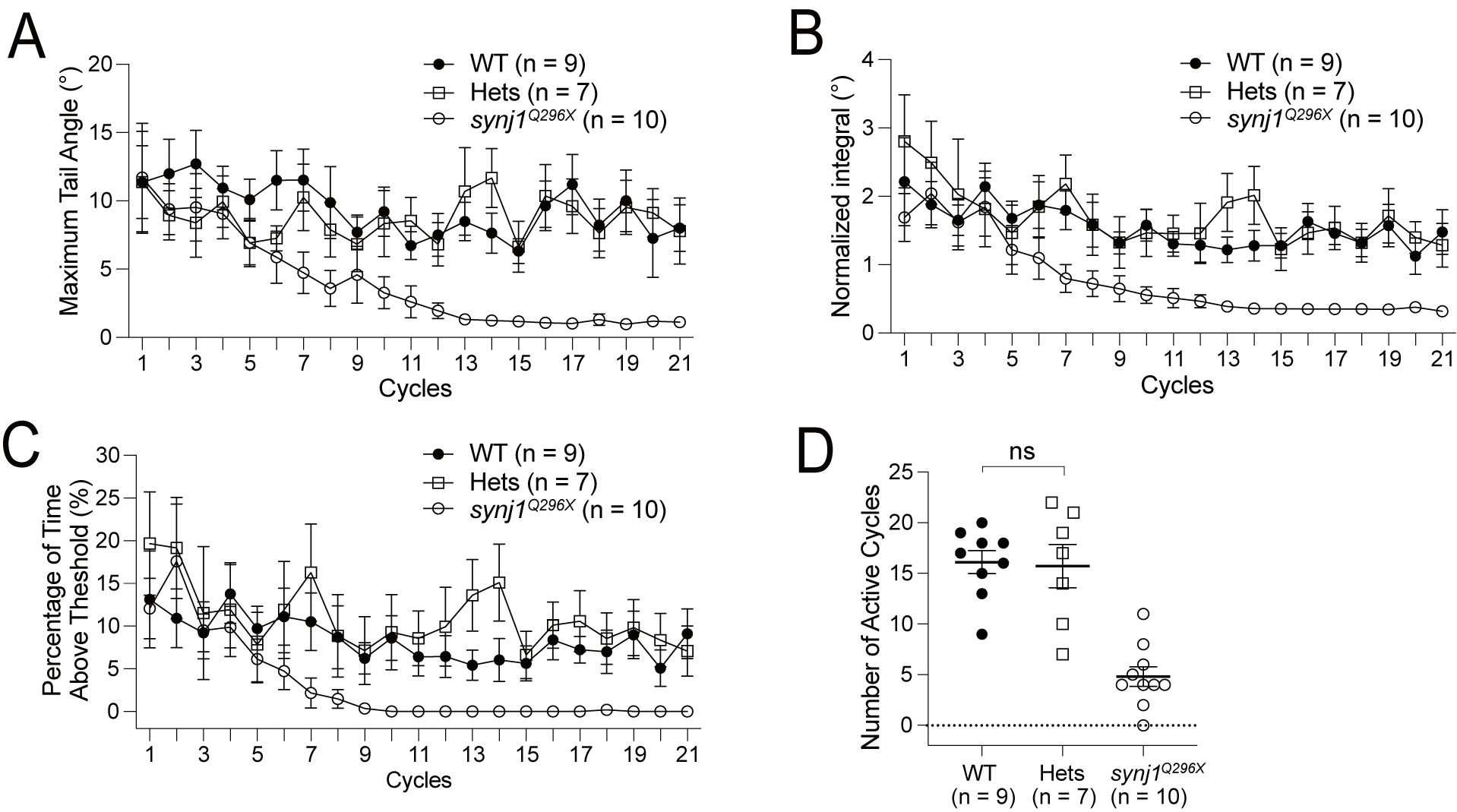

Supplement: Supplementary Figure 1 — Lack of a gene dosage effect in synj1Q296X sibling cohorts. Comparison of heterozygous synj1Q296X wild type siblings vs. homozygous wild type siblings does not reveal significant differences in performance. (A–D) The maximum tail angle, normalized integral, percentage of time above threshold, and number of active cycles. Mean ± SEM for each genotype was plotted and a two-way ANOVA with Benjamini-Hochberg correction was performed for the wild type larvae. Mutant data points were added for comparison. [file Image_1.tif]
