# Supplementary material for: Temporal Vestibular Deficits in synaptojanin 1 (synj1) Mutants
Source: Front Mol Neurosci. 2021 Jan 18;13:604189. doi: 10.3389/fnmol.2020.604189 (PMC7874208; doi:10.3389/fnmol.2020.604189)
Supplement: Supplementary file 3 [file Data_Sheet_1.PDF]

```
import json
import matplotlib.pyplot as plt
import pandas as pd
import numpy as np
import math
import os
import math

directory = '../ZZoutput/'  # Directory containing all the output
folders of ZebraZoom that you want to run this script on
threshActif = 5             # Threshold for the "Percentage of time
above threshold"
showResults = 0              # Put to 1 if you want to plot the
figures as the script goes through them
fps = 1277 / 42              # Frame per second videos where recorded
at

allVideoNames = []
for x in os.walk(directory):
  if len(x[0]) != len(directory):
    allVideoNames.append(x[0][len(directory):])

# allVideoNames = ['Mut2_2019-10-16-110849-0000before_poke']

variables = ['_centerFramePosition', '_previousBlackFramePosition',
'_centerFrameCyclePercentPosition', '_responseIntegral',
'_responseMaximum', '_responseTimeAboveThresh']
allDfColumns = np.array([[x + var for var in variables] for x in
allVideoNames]).flatten().tolist()
toFill = np.zeros((30, len(allDfColumns)))
df     = pd.DataFrame(toFill, columns = allDfColumns)

for idx, res in enumerate(allVideoNames):

  variables2    = ['tailAngle', 'medianTailAngle', 'tailAngle-
medianTailAngle']
  allDfColumns2 = np.array(variables2).flatten().tolist()
  toFill2       = np.zeros((1300, len(allDfColumns2)))
  df2           = pd.DataFrame(toFill2, columns = allDfColumns2)

  plt.figure(idx)

  with open(directory + res + '/results_' + res + '.txt') as f:
    supstruct = json.load(f)

  TailAngle_smoothed = np.array(supstruct['wellPoissMouv'][0][0][0]
['TailAngle_smoothed']) * (180/math.pi)
  median = np.transpose(pd.DataFrame(TailAngle_smoothed).rolling(5,
center=True).mean().values)[0]
  diff   = abs(TailAngle_smoothed - median)
```

```
  df2['tailAngle'][0:len(TailAngle_smoothed)]   = TailAngle_smoothed
  df2['medianTailAngle'][0:len(median)]         = median
  df2['tailAngle-medianTailAngle'][0:len(diff)] = diff

  df2.to_excel(res + ".xlsx")

  headX = supstruct['wellPoissMouv'][0][0][0]['HeadX']
  headY = supstruct['wellPoissMouv'][0][0][0]['HeadY']

  blackFramesList = [0]
  f2 = open(directory + res + '/blackFrames_' + res + '.csv', "r")
  for x in f2:
    if len(x):
      blackFramesList.append(int(x))
  f2.close()

  if True:
    blackFramesListOld = blackFramesList.copy()
    diffBlackFrames    = [(blackFramesList[idx+1] -
blackFramesList[idx]) for idx in range(1, len(blackFramesList)-1)]
    meanDiffBlack      = np.mean(diffBlackFrames)
    print("meanDiffBlack:", meanDiffBlack)
    addBlackInBetween  = [1 if diff > (meanDiffBlack * 1.5) else 0 for
diff in diffBlackFrames]
    blackFramesToAdd   = []
    for idx2, add in enumerate(addBlackInBetween):
      if add:
        blackFramesToAdd.append(blackFramesList[idx2+1] +
(blackFramesList[idx2+2] - blackFramesList[idx2+1])/2)
    for bToAdd in blackFramesToAdd:
      idx2 = 0
      while blackFramesList[idx2] < bToAdd:
        idx2 = idx2 + 1
      idx2 = idx2 - 1
      blackFramesList.insert(idx2+1, int(bToAdd))
    if len(blackFramesListOld) != len(blackFramesList):
      print(res,": Inserted", len(blackFramesList) -
len(blackFramesListOld), "black frames based on abnormally distant
subsequent black frames")
      print("Original list of black frames:", blackFramesListOld)
      print("Updated  list of black frames:", blackFramesList)

  responseCenter         = []
  previousBlackFrame     = []
  centerPercentPosition  = []
  responseIntegral       = []
  responseMaximum        = []
  percentTimeAboveThresh = []
```

```
  for idx3 in range(1, len(blackFramesList)):
    start  = blackFramesList[idx3-1]
    finish = blackFramesList[idx3]
    resCenter = 0
    resMax    = 0
    resInteg  = 0
    percTime  = 0
    for idx4 in range(start, finish):
      if not(math.isnan(diff[idx4])):
        resCenter = resCenter + idx4 * diff[idx4]
        resInteg  = resInteg  + diff[idx4]
        if diff[idx4] > threshActif:
          percTime = percTime + 1
        if diff[idx4] > resMax:
          resMax = diff[idx4]

    resCenter = resCenter / resInteg
    resInteg  = resInteg  / (finish - start)
    percTime  = (percTime  / (finish - start)) * 100

    previousBlackFrame.append(start)
    responseCenter.append(resCenter)
    centerPercentPosition.append((resCenter-start) / (finish-start+1))
    responseMaximum.append(resMax)
    responseIntegral.append(resInteg)
    percentTimeAboveThresh.append(percTime)

  df[res + '_centerFramePosition'][0:len(responseCenter)]
= responseCenter
  df[res + '_previousBlackFramePosition'][0:len(previousBlackFrame)]
= previousBlackFrame
  df[res + '_centerFrameCyclePercentPosition']
[0:len(centerPercentPosition)] = centerPercentPosition
  df[res + '_responseIntegral'][0:len(responseIntegral)]
= responseIntegral
  df[res + '_responseMaximum'][0:len(responseMaximum)]
= responseMaximum
  df[res + '_responseTimeAboveThresh'][0:len(percentTimeAboveThresh)]
= percentTimeAboveThresh

  fig, tabAx = plt.subplots(2, 1, figsize=(22.9, 8.8))

  xaxis = [(i/fps) for i in range(0, len(TailAngle_smoothed))]

  tabAx[0].plot(xaxis, TailAngle_smoothed, label='Tail Angle')
  tabAx[0].plot(xaxis, median,             label='Rolling Median of
Tail Angle')
  tabAx[0].scatter([(b/fps) for b in blackFramesList], [-0.57*(180/
math.pi) for b in blackFramesList], s = 60, c='k', marker='|', label =
'Black Frames')
```

```
  tabAx[0].set_xlabel('Time (seconds)')
  tabAx[0].set_ylabel('Degree (°)')
  tabAx[0].axis([0, len(TailAngle_smoothed)/fps, -0.6*(180/math.pi),
0.85*(180/math.pi)])
  tabAx[0].legend()

  tabAx[1].plot(xaxis, diff, label = '|Tail Angle - Rolling Median of
TA|', alpha = 0.5)
  tabAx[1].scatter([(b/fps) for b in blackFramesList], [-0.8 for b in
blackFramesList], s = 60, c='k', marker='|', label = 'Black Frames')
  tabAx[1].scatter([(r/fps) for r in responseCenter], responseMaximum,
s = 60, c='b', alpha = 1, label = 'Maximum')
  tabAx[1].scatter([(r/fps) for r in responseCenter],
responseIntegral, s = 60, c='r', alpha = 1, label = 'Normalized
Integral')
  tabAx[1].scatter([(r/fps) for r in responseCenter], [a/2 for a in
percentTimeAboveThresh], s = 60, c='g', label = 'Percentage of time
above '+str(threshActif) + '°')
  tabAx[1].axis([0, len(TailAngle_smoothed)/fps, -0.03*(180/math.pi),
0.85*(180/math.pi)])

  tabAx[1].set_xlabel('Time (seconds)')
  tabAx[1].set_ylabel('Degree (°)')
  def CtoF(x):
    return x * 2
  def FtoC(x):
      return x * 2
  secaxy = tabAx[1].secondary_yaxis('right', functions=(CtoF, FtoC))
  secaxy.set_ylabel('Percentage (%)')

  tabAx[1].legend()

  fig.suptitle(res, fontsize=16)
  plt.savefig(res + '.png')

  if showResults:
    plt.show()

  print("Analysis done for", res, "(video", idx+1, "out of",
len(allVideoNames), ")")
  print(" ")

df.to_excel("results.xlsx")
```
